# An investigation of the impact of futility analysis in publicly funded trials

**DOI:** 10.1186/1745-6215-15-61

**Published:** 2014-02-17

**Authors:** Benjamin GO Sully, Steven A Julious, Jon Nicholl

**Affiliations:** 1 Medical Statistics Group, School of Health and Related Research, University of Sheffield, 30 Regent Court, Regent Street, Sheffield S1 4DA, UK; 2 School of Health And Related Research, University of Sheffield, 30 Regent Court, Regent Street, Sheffield S1 4DA, UK

**Keywords:** Adaptive designs, Futility, Recruitment, Efficiency, Health technology assessment, Medical research council

## Abstract

**Background:**

Publicly funded trials regularly fail to recruit their target sample size or find a significant positive result. Adaptive clinical trials which may partly mediate against the problems are not often applied. In this paper we investigate the potential of a form of adaption in a clinical trial - a futility analysis - to see if it has potential to improve publicly funded trials.

**Methods:**

Outcome data from trials funded by two UK bodies, the Health Technology Assessment (HTA) programme and the UK Medical Research Council (MRC), were collected. These data were then used to simulate each trial with a single futility analysis using conditional power, undertaken after 50% to 90% of the patients had been recruited. Thirty-three trials recruiting between 2002 and 2008 met the inclusion criteria. Stopping boundaries of conditional powers of 20%, 30% and 40% were considered and outcomes included the number of trials successfully stopped and number of patients saved.

**Results:**

Inclusion of a futility analysis after 75% of the patients had been recruited would have potentially resulted in 10 trials, which went on to have negative results, correctly stopping for futility using a stopping boundary of 30%. A total of 807 patients across all the trials would potentially have been saved using these futility parameters. The proportion of studies successfully recruiting would also have increased from 45% to 64%.

**Conclusions:**

A futility assessment has the potential to increase efficiency, save patients and decrease costs in publicly funded trials. While there are logistical issues in undertaking futility assessments we recommend that investigators should aim to include a futility analysis in their trial design wherever possible.

## Background

Publicly funded trials in the United Kingdom (UK) have poor rates of recruitment [1,2], with only just over half successfully recruiting to their initial target sample size. A consequence of poor recruitment is that a trial can have reduced power. An underpowered trial may in turn be considered to be unethical, as the probability of finding a specified effect size in the trial would be so low that patients would enter into a trial which has little chance of achieving its objectives.

Trials with poor recruitment may also be more likely to request an extension to attempt to reach their target sample size. Between 2002 and 2008 nearly half of all publicly funded trials received an extension of some kind [1], with similar results found between 1994 and 2002 [2]. The extension may be either time-based, cost-based or both.

Innovative trial designs have been applied to avoid this problem in the pharmaceutical industry [3], where the main concerns include regulatory requirements, the need to get to market quickly and the necessity to develop only the most promising interventions [4]. Usually a pharmaceutical company would be undertaking trials to obtain sufficient evidence to get a license for a new treatment. As such, trials with negative results could mean a treatment does not get a license.

Publicly funded trials, such as those funded by the UK Medical Research Council (MRC) or National Institute for Health Research (NIHR) Health Technology Assessment programme (HTA), have different practical issues. For example, the trials may be assessing treatments which already are licensed but for which further information is required to get them used in practice. Valuable information is still obtained regardless of the final result [5]. Indeed, between 1993 and 2008 only 19% of superiority trials funded by the HTA programme had a statistically significant and clinically important result [6], with 76% of trials having results that were not statistically significant.

Interventions are often complex [6] in publicly funded trials. The outcomes are multi-faceted, often incorporating efficacy, quality of life and health economics, evaluating not just whether an intervention works but also if it offers value for money [5]. This increases the complexity of the whole trial. These practicalities, along with slow recruitment, can have a negative impact on trial duration [7-9].

Methods to improve the overall efficiency of clinical trials must account for the practical difficulties in undertaking the studies; it is the logistics and not the statistics which most impact a trial design [10]. Methods that are regularly applied in pharmaceutical trials may not be appropriate for publicly funded trials. Adaptive designs are one of the methods that are regularly applied in industry but have for the most part not been used in the public sector [11]. Principles such as sample size re-estimation, futility analysis and group sequential designs all have the potential to maximise efficiency and minimise costs in clinical trials. These methods have been shown to be statistically valid; it is often practical and expertise issues which prevent their use [11].

This paper will focus on futility assessment during a trial as a method for stopping trials which have low chances of finding a statistically significant result. We have applied existing conditional power methods for futility analysis retrospectively to the database of trials used by Sully *et al*. [1]. The results have been used to give an indication of the potential savings in patients, time and, by extension, costs that could be brought about by including futility analyses in publicly funded trials.

## Methods

### Data collection

Analysis was undertaken using the trial database created by Sully *et al*. [1] with some added restrictions to simplify interpretation. Trials were eligible if they met the following inclusion and exclusion criteria taken from Sully *et al*.:

1. They were multicentre.

2. Recruitment started on or after 1 January 2002.

3. Recruitment was originally planned to close on or before 31 December 2008.

4. They were not a cluster randomised trial.

Additional inclusion and exclusion criteria added were as follows:

1. They were a two-armed, parallel group superiority study.

2. The primary endpoint was binary or continuous.

3. They had evidence of a sample size calculation.

4. Data relating to the endpoint used in the sample size calculation were available at follow-up.

We restricted the studies to binary and continuous outcomes as in these cases it was always possible to determine the treatment effects in the trials and undertake calculations using summary data.

The existing database was sufficient for identifying most trials and further data extraction was undertaken to obtain the planned and observed effect sizes and type of endpoint for each trial. While utmost care went into ensuring that extracted data were correct there were occasional ambiguities in reporting; in these cases trial managers were contacted for clarification. In the event that no response was received, the trial was excluded from the study.

The types of data collected, split by endpoint (binary or continuous), are shown in Table 1. The aim was to collect the data to best allow for calculation of estimates of the conditional power and to inform the simulations: number of successes/failures in each group for binary outcomes and mean/standard deviation/sample size in each group for continuous outcomes. Unadjusted values were used for continuous outcomes.

**Table 1:** Characteristics of trials

	**Trial characteristics**	**Number (%)**	**Mean (SD)**	**Minimum - maximum**	**Successful (%)**
**Funding body**	**HTA**	15 (45.4)			4 (26.7)
	**MRC**	18 (54.5)			4 (22.2)
**Endpoint**	**Binary**	20 (60.6)			4 (20.0)
	**Continuous**	13 (39.4)			4 (30.8)
**Disease**	Cancer	2 (6.1)			0 (0.0)
**Area**	Mental health (including neurosciences/psychiatry/psychology)	5 (15.2)			3 (60.0)
	Orthopaedics/rheumatology (including back pain)	1 (3.1)			0 (0.0)
	Obstetrics & gynaecology	2 (6.1)			1 (50.0)
	Primary care	7 (21.2)			2 (28.6)
	Cardiology	3 (9.1)			0 (0.0)
	Gastroenterology	0 (0.0)			0 (0.0)
	Incontinence/urology	2 (6.1)			0 (0.0)
	HIV/AIDS	1 (3.0)			0 (0.0)
	Other	10 (30.3)			2 (20.0)
**Planned sample size**			751.4 (1426.9)	80 to 8,000	
**Final sample size**			615.2 (1452.1)	44 to 8,164	
**Power**			84.2 (5.2)	80 to 95	

HTA, Health Technology Assessment; MRC, Medical Research Council; SD, standard deviation.

Trials were also broken down into those which were ‘successful’ and those that were not. For this paper a successful trial is primarily defined as a trial finding a treatment effect greater than or equal to that specified in the sample size calculation; it was not necessary for this effect to be statistically significant. This is because in publicly funded trials it is not just important to see a statistically significant effect, but to see a worthwhile effect size; there are often many secondary endpoints, so the primary endpoint being non-significant does not mean the trial has failed to be worthwhile [5]. The final column of Table 1 gives the number of studies that were ‘successful’. The row for gastroenterology was included despite having zero numbers for consistency with the original paper where there were studies [11]. With respect to clinical area our data are too sparse to draw any conclusions.

As a secondary analysis the data were re-investigated with the requirement that the primary outcome must be statistically significant for a trial to be considered successful.

### Statistical methods

For each trial, a simulation was run using the planned sample size of the trial and observed number of successes (for binary trials) or effect size and standard deviation (for continuous trials). Trials were simulated using a binomial distribution or Normal distribution, as appropriate. The conditional power of the trial was then calculated at every 10% of the planned sample size (up to the final sample size, if final recruitment was below that planned), as well as after 75%, using the methods shown below. Each simulation was repeated 50,000 times. The mean conditional power was calculated at each proportion of the sample size for each trial and used to judge whether a trial should be stopped for futility using a boundary, *γ*, of either *γ* = 0.2, *γ* = 0.6 or *γ* = 0.4. Thus, for the first boundary value, if the conditional power is less than 0.2, for instance, then the study will stop early for futility.

When calculating the conditional power of a trial an assumption must be made about the distribution of the remainder of the trial data. Three common choices are that it will follow the null hypothesis, the alternative hypothesis or the observed trend so far [12]. In this paper, we assume the data will follow the alternative hypothesis. This gives trials the least chance of stopping early, and so maximises information. The conditional power was calculated using the following result [12]:

$$\mathrm{CP}=1-\Phi \left\{\frac{Z_{1-\alpha /2}-\left(Z_{t}\sqrt{\tau }+\left(Z_{1-\frac{\alpha }{2}}+Z_{1-\beta }\right)\left(1-\tau \right)\right)}{\sqrt{1-\tau }}\right\}$$

where Φ is the cumulative density function of the standard Normal distribution, *Ζ* is the standard Normal random variable, *Ζ*_*t*_ is the *Ζ*-score at interim, *τ* is the proportion of patients recruited, and *α* and 1-*β* are the planned type I and II errors, respectively. Conditional power was calculated on a two-sided basis, so the conditional power was the probability of finding either a significant positive or negative result.

In our analysis the assumption is that only one assessment of futility is planned in the study.

We also make the additional assumption that we have all patients recruited available who have been recruited for the futility assessment. The implications of this assumption will be discussed later.

As highlighted, one of the assumptions is that the remainder of the trial will follow the alternative hypothesis. Thus, the estimate of effect will be taken from that used in the sample size calculation. If the effect in this original calculation was overstated then the conditional power will be low and a trial will stop for futility even when a possibly clinically meaningful difference has been observed [13].

The results in this paper are particularly sensitive to this assumption as we define ‘success’ as observing the pre-specified estimate of effect.

## Results

**Figure 1:**
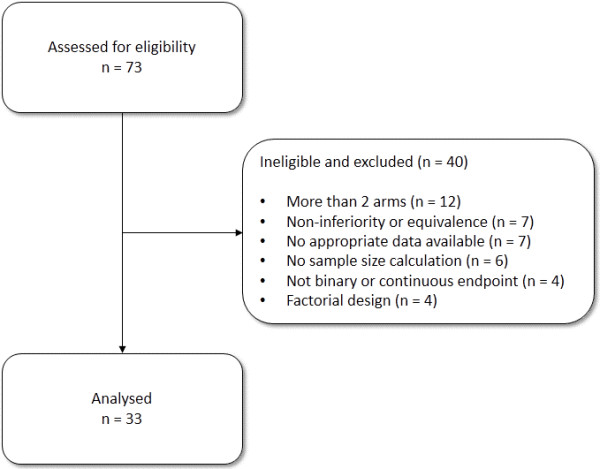
Flow of trials through the study.

The seventy-three trials from the previous database [1] were assessed for eligibility; Figure 1 shows the flow of these trials through the study. Thirty-three (45%) of the trials were eligible; the main reasons for ineligibility were a trial having more than two arms (12; 16%), the main trial objective being non-inferiority or equivalence (7; 10%), the trial not having appropriate data available (7; 10%) or not having a power calculation (6; 8%). Characteristics of eligible trials are shown in Table 1. Eight of the trials were successful (24%). This result is consistent with Dent and Raftery [6] who used a definition of statistically significant (and observed 26% of trials) and statistically significant with clinically meaningful effects (and observed 19% of trials).

**Figure 2:**
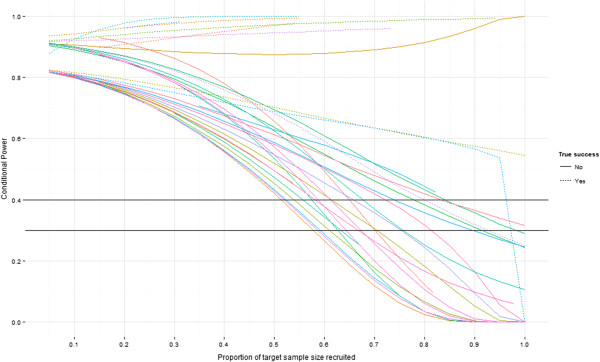
**Conditional power of each trial as simulated patients are recruited.** Black lines indicate 30% and 40% conditional power boundaries.

Figure 2 shows the average conditional power of each trial from the simulations. Trials are broken down into those that were successful and those that were not using our primary definition of successful. One trial had low conditional power towards the end of recruitment despite finding an effect larger than planned (relatively): this is due to the lack of statistical significance found by the trial, that is *P*-values of *P* = 0.050 [7]. This was due to the observed response rates being different than assumed.

Conversely, one trial had very high average conditional power despite finding an effect much smaller than originally planned; this is because the trial undertook a sample size re-estimation and went on to recruit far more than its original planned sample size – based on a new effect size - eventually finding statistically significant evidence but one that was smaller than used in the original sample size calculation [14].

The majority of trials which eventually ‘fail’ appear to have conditional power below 40% by the time 70% of patients have been recruited, and below 30% by the time 80% of patients have been recruited. This is summarised in Table 2, where results for ‘true success’ versus ‘stop due to conditional power’ are given for every 10% of patients after 50% (and at 75% of target recruitment), and for both 30% and 40% conditional power boundaries. In each of these tables higher numbers in the off-diagonals are desirable, as this indicates that either: 1) trials have found a successful result and would not have been stopped due to futility, had an analysis been done; or 2) trials have not found a successful result and would have been stopped due to futility.

**Table 2:** Number and percentage of trials stopping for futility after 50%, 60%, 70%, 75%, 80% or 90% of patients recruited based on stopping boundaries of 20%, 30% or 40%

	**Number (percentage) of trials**			**True successful result**					
				**CP stopping boundary 20%**		**CP stopping boundary 30%**		**CP stopping boundary 40%**	
				**Successful trial**		**Successful trial**		**Successful trial**	
				**Yes**	**No**	**Yes**	**No**	**Yes**	**No**
**Stop if futility analysis done**	50% of patients	Stopped early	Yes	0	0	0	0	0	0
			No	8 (28%)	21 (72%)	8 (28%)	21 (72%)	8 (28%)	21 (72%)
	60% of patients	Stopped early	Yes	0	0	0	3 (12%)	0	8 (32%)
			No	5 (20%)	20 (80%)	5 (20%)	17 (68%)	5 (20%)	12 (48%)
	70% of patients	Stopped early	Yes	0	5 (21%)	0	11 (46%)	0	12 (50%)
			No	5 (21%)	14 (58%)	5 (21%)	8 (33%)	5 (21%)	7 (29%)
	75% of patients	Stopped early	Yes	0	7 (30%)	0	10 (43%)	0	14 (61%)
			No	4 (17%)	12 (52%)	4 (17%)	9 (39%)	4 (17%)	5 (22%)
	80% of patients	Stopped early	Yes	0	9 (41%)	0	11 (50%)	1 (5%)	14 (64%)
			No	4 (18%)	9 (41%)	4 (18%)	7 (32%)	3 (14%)	4 (18%)
	90% of patients	Stopped early	Yes	0	11 (55%)	0	12 (60%)	1 (5%)	15 (75%)
			No	4 (20%)	5 (25%)	4 (20%)	4 (20%)	3 (15%)	1 (5%)

CP, conditional power.

It is important to avoid false negatives – in this case, higher numbers in the top-left cell of the tables – as this indicates a trial has been stopped due to futility, when it would have been successful had it continued. One trial could be considered to have incorrectly stopped for futility in our analysis (Table 2). This study found a statistically significant result but a very small effect size, so it is classified as unsuccessful by our definitions. This trial would have been stopped for futility after 80% or more of patients had been recruited using a 40% stopping boundary.

The analysis was repeated using our secondary definition of a successful trial – that the trial has a ‘statistically significant’ outcome. Ten (30%) of the trials were considered successful using this classification compared to eight (24%) using the previous definition; this is higher because of statistically significant results for effects smaller than planned on the sample size. Table 3 shows the same summary as Table 2 using this alternative classification.

**Table 3:** Number and percentage of trials stopping for futility when requiring a statistically significant result to consider a trial ‘successful’

	**Number (percentage) of trials**			**True successful result**					
				**CP stopping boundary 20%**		**CP stopping boundary 30%**		**CP stopping boundary 40%**	
				**Successful trial**		**Successful trial**		**Successful trial**	
				**Yes**	**No**	**Yes**	**No**	**Yes**	**No**
**Stop if futility analysis done**	50% of patients	Stopped early	Yes	0	0	0	0	0	0
			No	9 (31%)	20 (69%)	9 (31%)	20 (69%)	9 (31%)	20 (69%)
	60% of patients	Stopped early	Yes	0	0	0	3 (12%)	1 (4%)	7 (28%)
			No	6 (24%)	19 (76%)	6 (24%)	16 (64%)	5 (20%)	12 (48%)
	70% of patients	Stopped early	Yes	0	5 (21%)	0	11 (46%)	0	12 (50%)
			No	5 (21%)	14 (58%)	5 (21%)	8 (33%)	5 (21%)	7 (29%)
	75% of patients	Stopped early	Yes	0	7 (30%)	0	10 (43%)	0	14 (61%)
			No	4 (17%)	12 (52%)	4 (17%)	9 (39%)	4 (17%)	5 (22%)
	80% of patients	Stopped early	Yes	0	9 (41%)	0	11 (50%)	0	15 (68%)
			No	4 (18%)	9 (41%)	4 (18%)	7 (32%)	4 (18%)	3 (14%)
	90% of patients	Stopped early	Yes	0	11 (55%)	0	12 (60%)	0	16 (80%)
			No	4 (20%)	5 (25%)	4 (20%)	4 (20%)	4 (20%)	0

CP, conditional power.

It is worth noting that for both Table 2 and Table 3 there are a different number of studies which recruit sufficient patients to get to 50%, 60%....90% of target sample size [1], falling from 29 studies that achieved 50% of their target sample size to 20 that reached 90%.

For our analysis we are concerned with the overall proportion of trials, particularly how a futility assessment will impact on the proportion of trials that successfully recruit to their target sample size. It is worth highlighting, however, that trials that continue after a futility assessment will not necessarily go on to be successful. With a futility assessment after 70%, 80% and 90% of patients, 62% (8 of 13), 64% (7 of 11) and 50% (4 of 8), respectively, would still go on to be unsuccessful according to Table 3 with a 30% conditional power boundary (it should be noted that these numbers are based on a small number of trials). We did not consider minimising the proportion of trials which go on to be unsuccessful which would be the case with higher conditional power at a futility assessment.

Table 4 shows the potential saving in patients across all 33 trials had a futility analysis been undertaken. Maximum savings are found 75% of the way through trials using a 20% boundary (634 patients), a *γ* = 0.30 boundary (807 patients) and a *γ* = 0.40 boundary (1,390 patients).

**Table 4:** Number of patients potentially saved

**Number of patients saved (after inflation in sample size)**		**Stopping boundary**		
		**20%**	**30%**	**40%**
**Proportion of patients recruited**	50%	0	0	0
	60%	0	325	751
	70%	342	686	1,027
	75%	634	807	1,390
	80%	512	667	1,145
	90%	467	570	711

Numbers and percentages of trials considered to have ‘successfully recruited’ after undertaking a futility analysis are given in Table 5. For this table we have coded a trial as successfully recruited to target if it stops for futility, regardless of whether the trial then went on to meet its original target sample size.

**Table 5:** Number and percentage of trials classed as ‘successfully recruiting’ based on stopping boundaries of 30% or 40%

**Number (percentage) of trials successfully recruiting**	**‘Successful’ recruitment**					
	**CP stopping boundary 20%**		**CP stopping boundary 30%**		**CP stopping boundary 40%**	
	**Yes**	**No**	**Yes**	**No**	**Yes**	**No**
**50% of patients**	15 (45%)	18 (55%)	15 (45%)	18 (55%)	15 (45%)	18 (55%)
**60% of patients**	15 (45%)	18 (55%)	17 (52%)	16 (48%)	21 (64%)	12 (36%)
**70% of patients**	19 (58%)	14 (42%)	20 (61%)	13 (39%)	21 (64%)	12 (36%)
**75% of patients**	19 (58%)	14 (42%)	21 (64%)	12 (36%)	21 (64%)	12 (36%)
**80% of patients**	19 (58%)	14 (42%)	20 (61%)	13 (39%)	20 (61%)	13 (39%)
**90% of patients**	19 (58%)	14 (42%)	19 (58%)	14 (42%)	19 (58%)	14 (42%)

CP, conditional power.

Undertaking a futility analysis after 50% of patients have been recruited leaves the proportions unchanged: 15 (45%) of the trials are classed as successfully recruiting. This proportion is highest (21 trials; 64%) when the futility analysis is undertaken after 70% or 75% of patients are recruited. It should be noted that because some of the trials failed to ever reach higher proportions of their target sample size (80% or 90%) they would not have had chance to undertake a futility assessment.

## Discussion

We recommend that a futility assessment be considered for all trials and in particular for publicly funded trials. With a futility assessment there is the potential to considerably increase the proportion of trials that successfully recruit to the target sample size. There would need to be a small inflation in the target sample size but this would be offset by the savings made from the trials stopping early.

Including a futility assessment can increase the chances of a study successfully recruiting. In our analysis, with a futility assessment after 75% of the target sample size has been recruited, the proportion of studies successfully recruiting can be increased from 45% to 64%.

It should be noted, however, that a futility assessment will not ensure the trial goes on to be successful. In our analysis with a futility assessment after 75% of patients with conditional power of 30% the majority of trials - 69% (9 of 13) - would still go on to be unsuccessful. Altering the conditional power at the futility assessment will have an impact on the proportion of trials that go on to be successful.

In our study, only 24% of trials were successful by our definition which was consistent with the work of Dent and Raftery using slightly different definitions [6]. If the prevalence of successful trials is different, then the potential savings in terms of sample sizes will vary. It is likely that the chance of a successful trial will vary by disease area. However, in our study the data are too sparse to draw any firm conclusions.

A balance must be struck when undertaking a futility analysis earlier in a trial, which allows for greater opportunity for savings in the sample size compared to undertaking a futility assessment later when a trial is more likely to be correctly designated as futile. In our analysis, we found that a futility assessment after at least 70% of the target sample size has been recruited maximised the chances of stopping for futility with little chance of stopping after 50% of the target sample size. Our results are consistent with those of Lachin [15]. Assuming the remainder of the trials followed the alternative hypothesis, thus author's study found that if there was no difference observed in the trial the conditional power would still be above 25% after 50% of patients (and a little short of 30%).

The issue of early futility assessment can be illustrated with simple calculations. Imagine a conventional sample size calculation where the sample size, n, is estimated for a clinically significant difference, d, for 90% power and a two-sided significance level, 5% [16]. Suppose half way through a naïve power calculation was done taking the simple mean of the observed effect for the first 50% of patients and the anticipated effect for the remainder of the trial, d (and standard deviation, *σ*). If the observed effect was zero (with the standard deviation observed, s, the same as *σ*) then the simple average would be d/2 and the naïve power calculation would be 37%. Thus, half way through the trial, the effect size (assuming other assumptions used in the sample size are consistent, such as the estimate of the population standard deviation) can be small and the study may still not stop for futility. Futility assessments are not without their downsides. Introducing an extra chance to stop the trial due to lack of a positive result will increase the Type II error β up to a maximum of [12]

$$\max\left(\beta_{\text{final}}\right)=\frac{\beta_{\text{planned}}}{1-\gamma }$$

(equivalent to a decrease in power from 90% to 85.7% for *γ* = 0.30); however, this maximum is only reached if the trial is continuously monitored for futility [12]. Chang and Stein [17], highlight, though, that with a futility assessment the Type I error also is reduced which,when accounted for, offsets the need to inflate sample size due to a loss of power.

Conservatively, we can ignore the effect on the Type I error and adjust the sample size to account for the impact on the power. It has been suggested that for one futility analysis the associated decrease in power is relatively small provided the boundary *γ* is less than or equal to 40% [17]. Using a boundary of this size, power is decreased to a minimum of 97% of the previous value, so the sample size of a 90% powered study should be inflated by a maximum of 10% to account for this loss in power.

It should be noted, however, that the reduction in power will depend both on the timing of the futility assessment and the conditional power boundary used, and will usually be much lower than this. For example, assessing futility after 75% of patients are recruited using a 30% boundary will only inflate the sample size of a 90% powered study by approximately 6%, and this would be less if the futility assessment is earlier. Investigators can determine this inflation themselves using available statistical software [18] with appropriate stopping boundaries [19].

Although there needs to be an adjustment in the maximum planned sample size when designing the study, the number of patients who are recruited into the study may well be considerably less than this maximum. This is because, as we have observed in this paper, if the null hypothesis is true the actual sample size would be considerably smaller as the study would stop for futility.

It should be noted that the methods applied and results obtained in this paper assume that the time between recruitment and primary outcome measurement (*t*) is very small relative to the total time spent recruiting (*T*), that is, t/T is small. This assumption means that when assessing futility after *n*_0_ evaluable patients have been recruited, outcome data will be available on all of them. However, if this assumption does not hold and t/T is not small there will be those who have been recruited to the trial but do not have evaluable data for the futility assessment. These patients are known as pipeline patients [20].

We observed that the optimum time to undertake a futility analysis is after 75% of patients of the target sample size have been recruited. In actuality, this is 75% of patients for whom we have an assessment at the relevant endpoint. Hence, a futility assessment requiring 75% of evaluable patients may now encounter the problem of having fully recruited at the time of the assessment. Denoting the number of patients recruited (evaluable or not) by *n*_*r*_ and the planned sample size by *N*, in this case we find that

$$n_{r}=\left(0.75+\frac{t}{T}\right)N$$

and a successful futility assessment will only save

$$\left(1-\left(0.75+\frac{t}{T}\right)\right)N$$

patients. Clearly if *t/T ≥*0.25 then there is little benefit to be gained from a futility assessment as the trial will have fully recruited. The results found in this paper are, therefore, indicative of the maximum savings attained if the time between recruitment and outcome is very short relative to the total recruitment time of the trial, such as, for example, trials in emergency medicine [21].

In cases where the follow-up time for the primary outcome is relatively long, alternative methods must be considered. Hampson and Jennison discuss this situation in detail, proposing methods for delayed responses which include analysis of pipeline data [20].

The implication of what we have highlighted is that the potential reductions in the sample size highlighted in Table 4 are maximum potential sample size reductions, achievable in some areas where the primary outcome is at an early time point but less so in other areas. A further issue is that the impact of futility depends on the proportion of trials where the null hypothesis is true. This is unknown and will vary from trial to trial.

There are many benefits to including a futility analysis in a trial. Stopping a futile trial early allows patients who would otherwise have been assigned to an inferior treatment to instead receive the best possible care. Additionally, with only a finite capacity for research the patients, no longer being recruited into a stopped trial, could go on to take part in other, more promising studies. Additionally, investigators can focus the time saved on further research rather than completing a negative trial.

## Conclusions

A futility assessment in a clinical trial has the potential to increase efficiency, save patients, and decrease the costs of publicly funded trials. While there are logistical issues in undertaking a futility assessment, whenever possible investigators should aim to include a futility analysis in their trial design, with the results from the 33 trials in the paper suggesting maximum savings found by doing so when 75% of the target sample has been recruited, using a boundary of *γ* = 0.3.

## Abbreviations

CP: conditional power; HTA: Health technology assessment; MRC: Medical Research Council; NIHR: National Institute for Health Research.

## Competing interests

The authors declare that they have no competing interests.

## Authors’ contributions

BGOS carried out the data extraction, simulations, analysis and report writing. SAJ conceived the study, assisted with interpretation of results and reporting. JN assisted in interpretation and contributed towards report writing. All authors read and approved the final manuscript.
